# Supplementary material for: Reinforcement learning control of a biomechanical model of the upper extremity
Source: Sci Rep. 2021 Jul 14;11:14445. doi: 10.1038/s41598-021-93760-1 (PMC8280157; doi:10.1038/s41598-021-93760-1)
Supplement: Supplementary file 1 — Supplementary Information 1 [file 41598_2021_93760_MOESM1_ESM.pdf]

# Reinforcement Learning Control of a Biomechanical Model of the Upper Extremity

**Florian Fischer[1,*], Miroslav Bachinski[1], Markus Klar[1], Arthur Fleig[1], and Jörg Müller[1]**

[1]University of Bayreuth, Bayreuth, Germany
[*]florian.j.fischer@uni-bayreuth.de

## Supplementary information

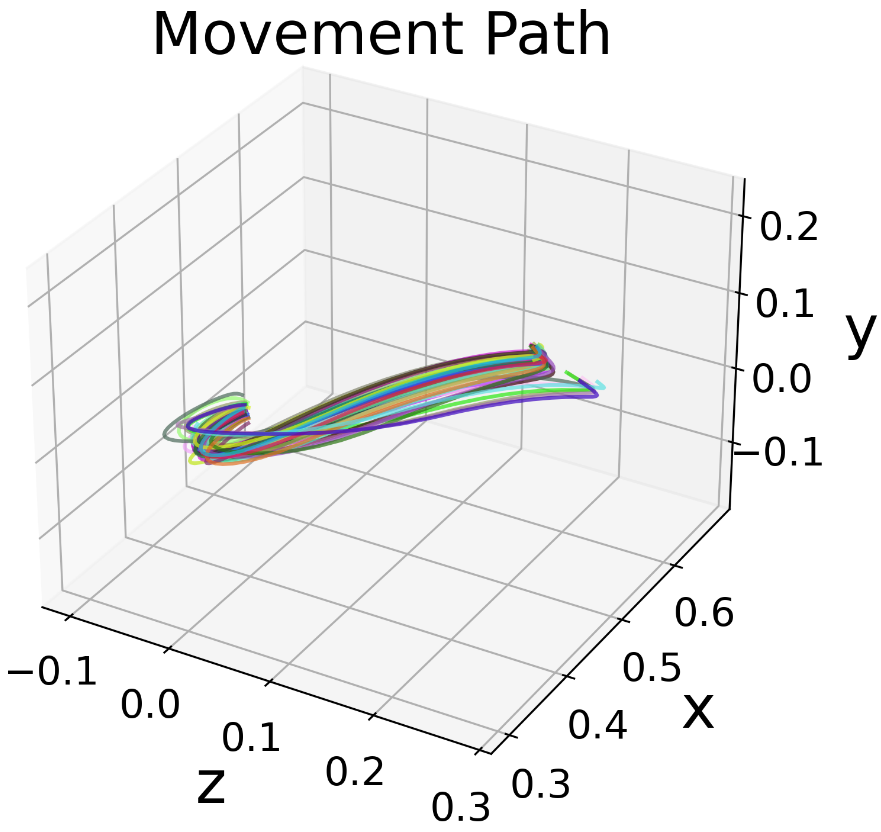


**Supplementary Figure S1. Movement with change of direction (3D Path).** For some trajectories, the direction changes towards the end of the movement (here: ID 2, 35cm distance, movements between targets 5 and 6).

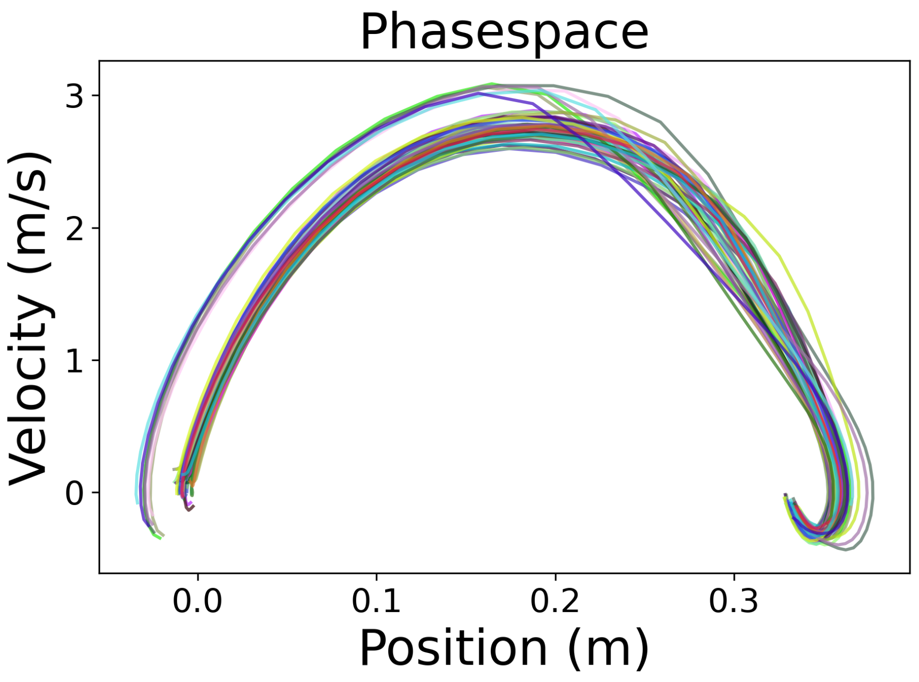


**Supplementary Figure S2. Movement with change of direction (Phasespace).** Changes of direction towards the end of a movement are also visible in the appendix at the right side of the Phasespace plot. (here: ID 2, 35cm distance, movements between targets 5 and 6).

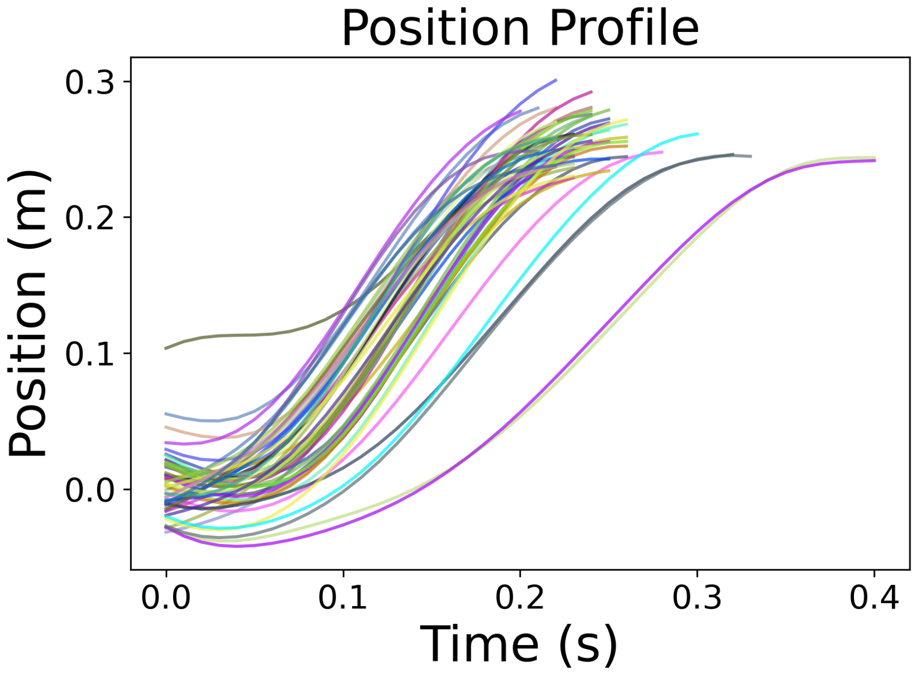


**Supplementary Figure S3. Trajectory variability in ID 1 movements (Position).** For simple ID 1 movements, the between-trial variability is the largest. In addition, some "outliers" with considerably higher reaction time might occur (here: ID 1, 25cm distance, movements between targets 1 and 2).

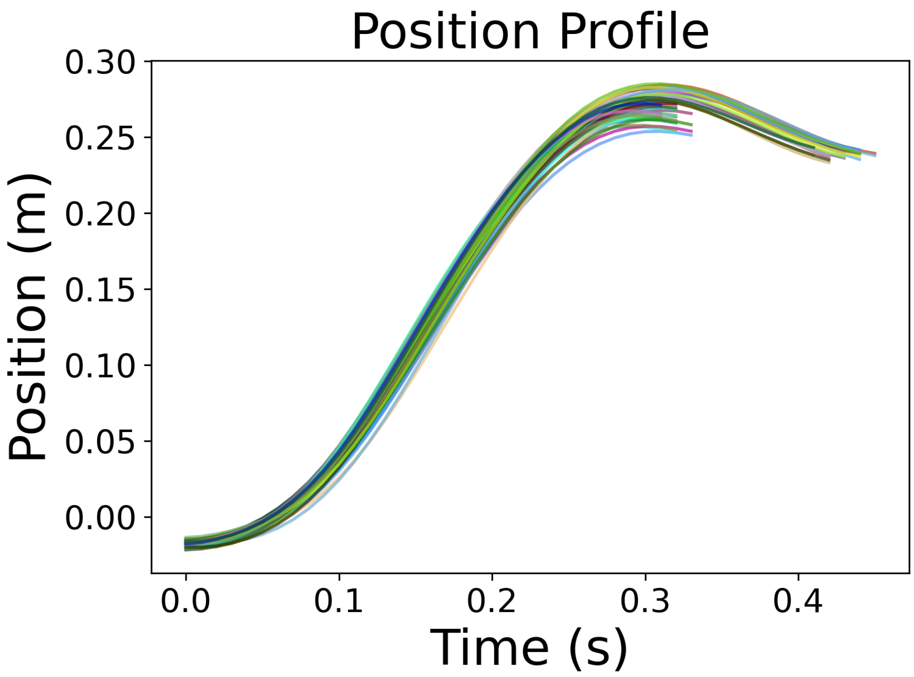


**Supplementary Figure S4. Corrective Submovements (Position).** For some trials, the end-effector does not stay inside the target for the required 100ms. A corrective submovement (here after 0.3s) then enables a second attempt (here: ID 2, 25cm distance, movements between targets 9 and 10).

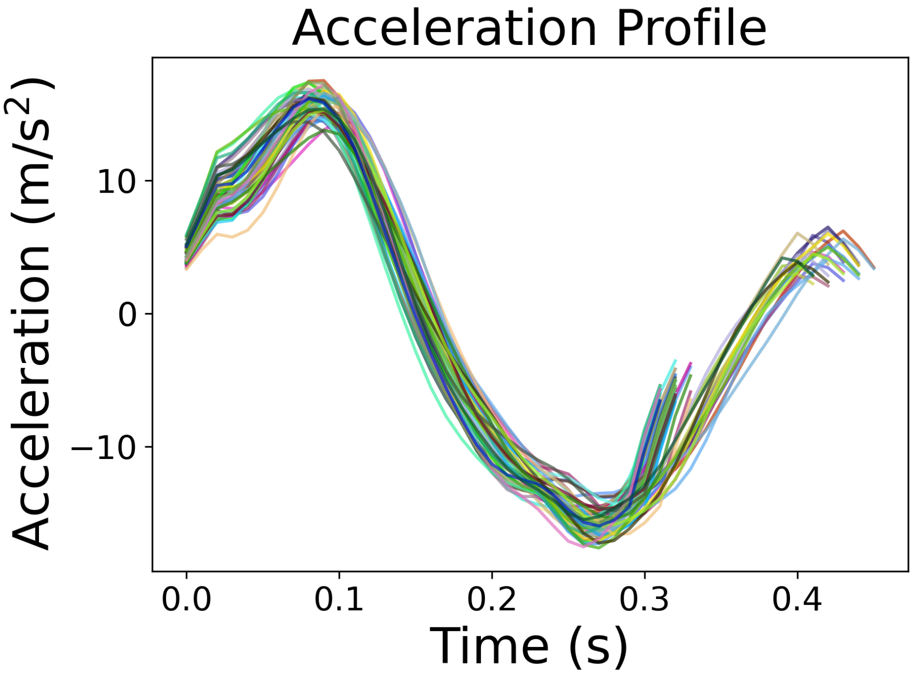


**Supplementary Figure S5. Corrective Submovements (Acceleration).** For some trials, the end-effector does not stay inside the target for the required 100ms. A corrective submovement (here after 0.3s) then enables a second attempt (here: ID 2, 25cm distance, movements between targets 9 and 10).

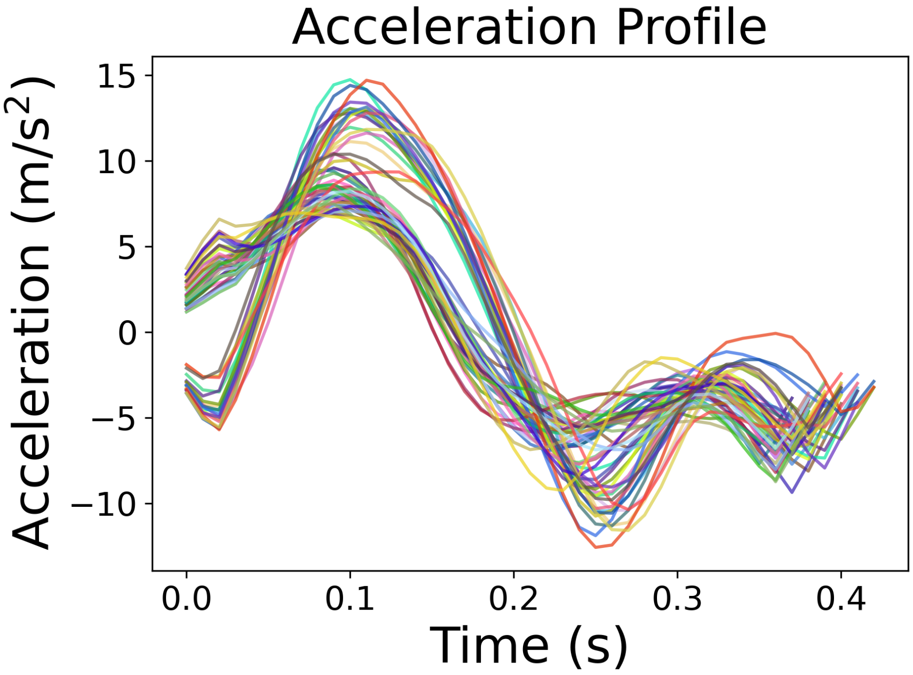


**Supplementary Figure S6. Movements with different initial conditions (Acceleration).** The extent of corrective sub-movements in the previous movement (see Supplementary Fig. S4 and S5) determines the initial acceleration, which in turn affects the shape of, e.g., the acceleration profile (here: ID 2, 25cm distance, movements between targets 10 and 11).

| Distance $D$ | Width $W$ | ID |
|---|---|---|
| 0.05 | 0.05 | 1 |
| 0.15 | 0.15 | 1 |
| 0.25 | 0.25 | 1 |
| 0.15 | 0.05 | 2 |
| 0.25 | 0.0833 | 2 |
| 0.35 | 0.1167 | 2 |
| 0.15 | 0.0214 | 3 |
| 0.25 | 0.0357 | 3 |
| 0.35 | 0.05 | 3 |
| 0.35 | 0.0233 | 4 |

**Supplementary Table S1. Conditions in the Fitts' Law Type Task.** Distance between initial point and target $D$ (in meters), diameter of target sphere $W$ (in meters), and resulting Index of Difficulty (ID) of task conditions used in the Fitts' Law type task.
